# Supplementary material for: Post Mortem Study on the Effects of Routine Handling and Manipulation of Laboratory Mice
Source: Animals (Basel). 2022 Nov 22;12(23):3234. doi: 10.3390/ani12233234 (PMC9737896; doi:10.3390/ani12233234)
Supplement: Supplementary file 1 [file animals-12-03234-s001.zip › animals-2029957-supplementary.pdf]

Post mortem Study on the Effects of Routine Handling and Manipulation of Laboratory Mice

Charles-Antoine Assenmacher, Matthew Lanza, James Carmine Tarrant, Kristin Lee Gardiner,

Eric Blankemeyer, Enrico Radaelli

Supplementary material:

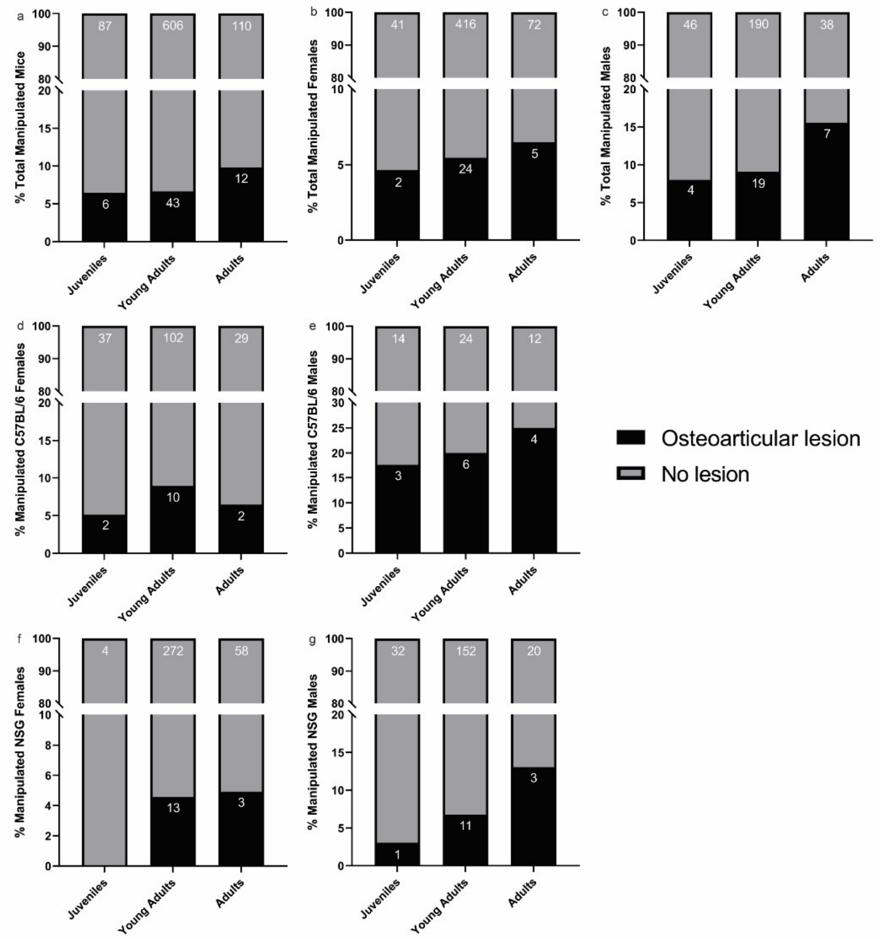

Figure S1: Statistical comparisons of the frequency of osteoarticular traumatic lesion in manipulated mice across different age categories.

Statistical comparisons of: (a) all manipulated mice across the different age categories (P= 0.2916 (ns)), (b) all manipulated female mice across the different age categories (P= 0.6526 (ns)), (c) all manipulated male mice across the different age categories (P= 0.2292 (ns)), (d) all manipulated female C57BL/6 mice across the different age categories (P= 0.7820 (ns)), (e) all manipulated male C57BL/6 mice across the different age categories (P= 0.6037 (ns)), (f) all manipulated female NSG mice across the different age categories (P= 0.8003 (ns)), (g) all manipulated male NSG mice across the different age categories (P= 0.1538). ns $P > 0.05$; * $P \leq 0.05$; ** $P \leq 0.01$ by Chi-square test for trend. ns, no significance.

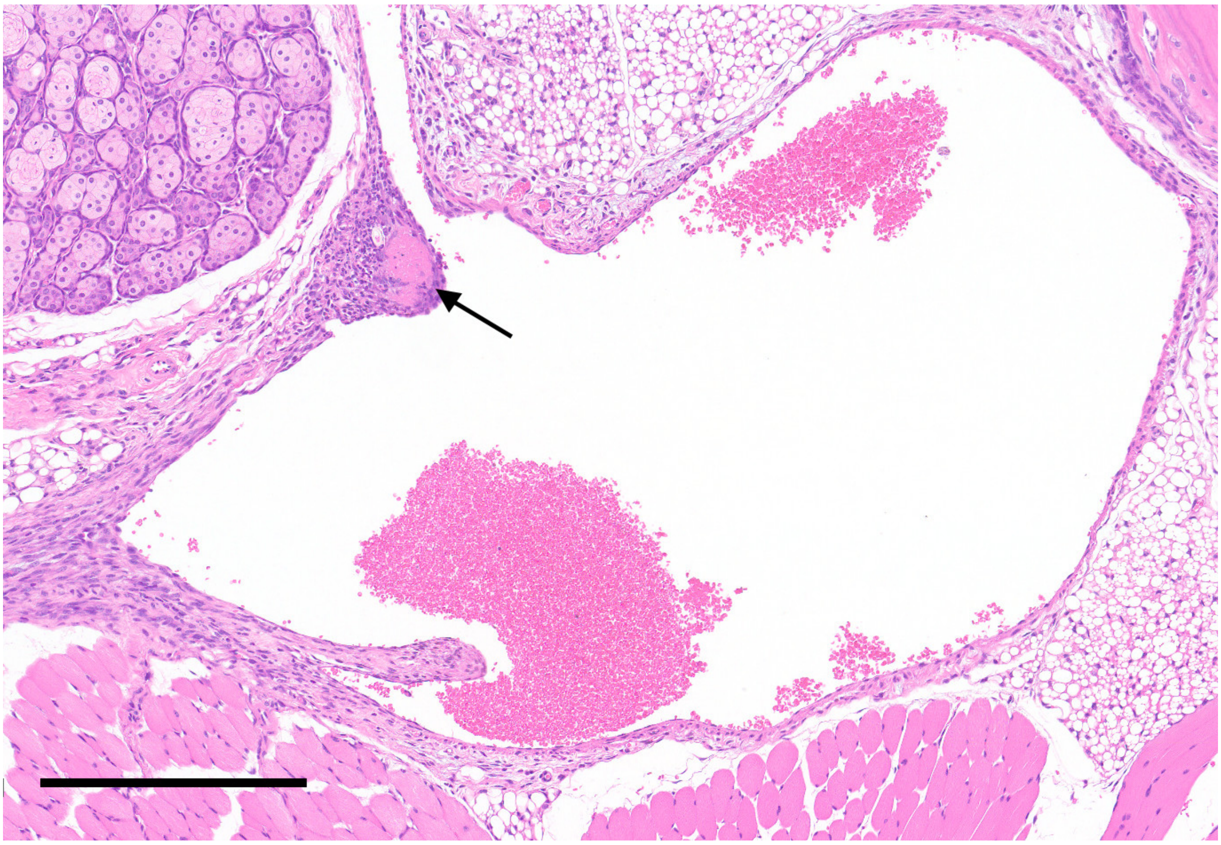

Figure S2: Non-occlusive mural thrombus associated with facial vein puncture.

Photomicrograph of the facial vein showing a non-occlusive mural fibrin thrombus with an intralesional hair shaft (black arrow). H&E stain. Scale bar, 300mm

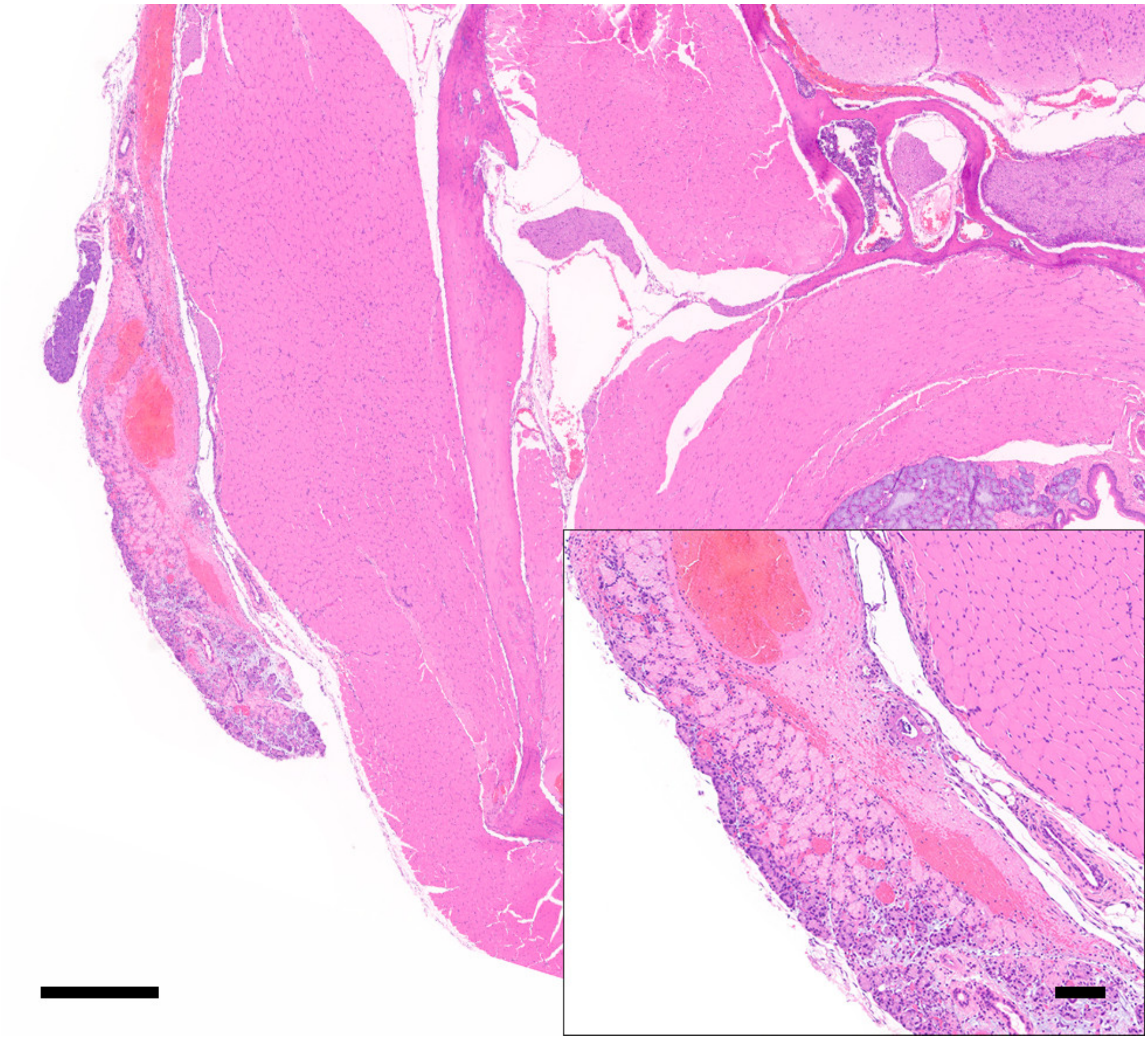

Figure S3: Extensive salivary gland necrosis associated with facial vein puncture.

Photomicrograph of the parotid salivary gland. The gland is extensively necrotic with hemorrhage. H&E stain. Left scale bar, 500mm; Inset scale bar, 100mm.

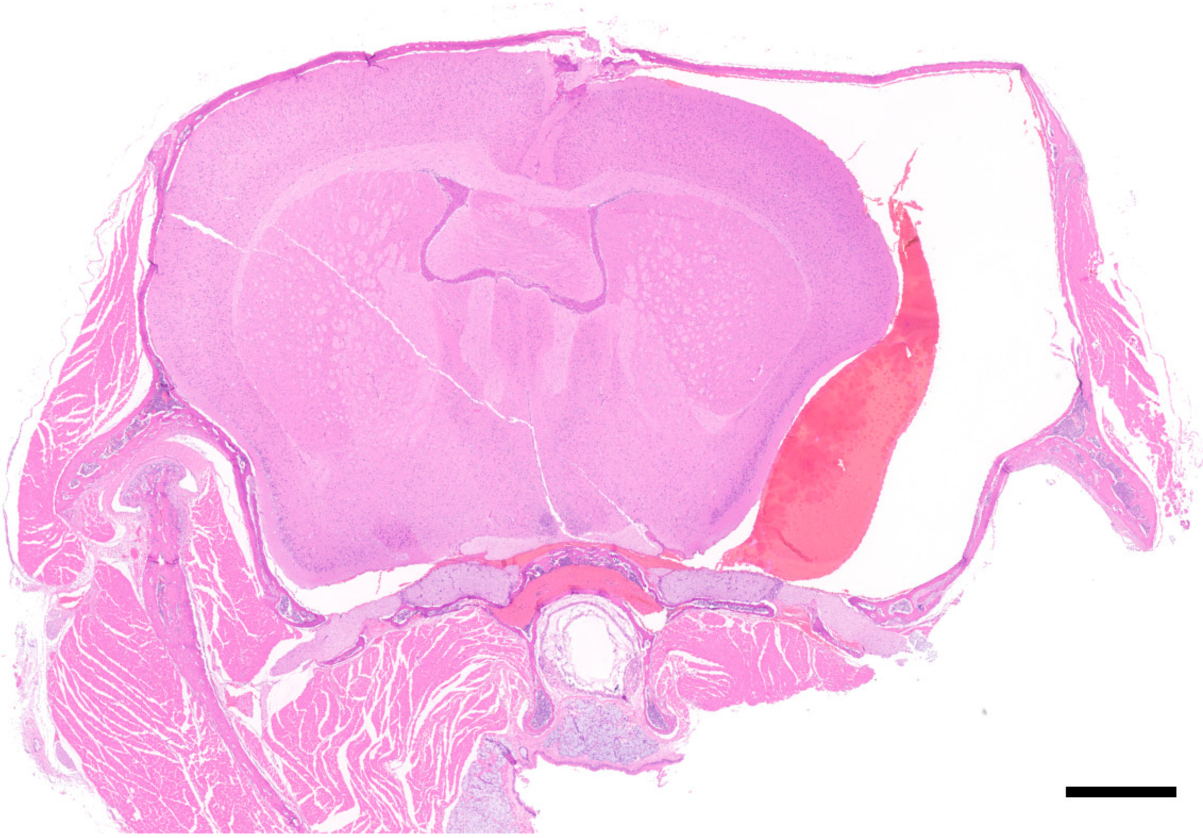

Figure S4: Intracranial hemorrhage associated with facial vein puncture.

The subdural space is unilaterally severely expanded by acute hemorrhage. The cause of death in this mouse was attributed to intracranial hemorrhage secondary to accidental perforation of the skull during facial vein blood collection, which was performed a few hours prior to death. H&E stain. Scale bar, 1mm.

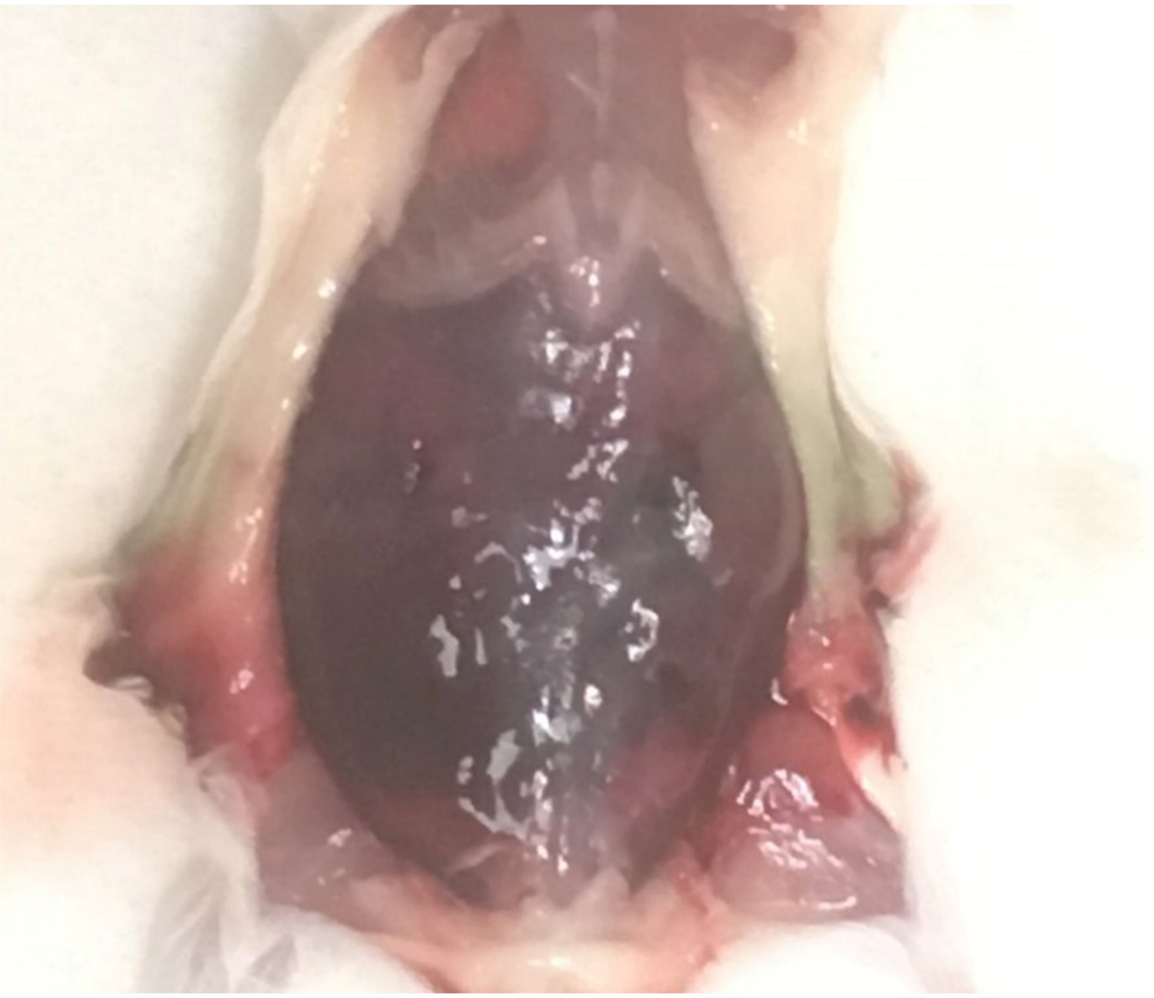

Fig. S5: Hemoabdomen following IP injection.

Macroscopic image of a mouse with severe hemoabdomen. The cause of death in this mouse was attributed to the hemoabdomen secondary to the IP injection, which was performed a few hours prior to death.
